# Periodontal Pathogens: A Crucial Link Between Periodontal Diseases and Oral Cancer

**DOI:** 10.3389/fmicb.2022.919633

**Published:** 2022-06-30

**Authors:** Tian-Jiao Li, Yi-hang Hao, Ya-ling Tang, Xin-hua Liang

**Affiliations:** ^1^State Key Laboratory of Oral Diseases and National Clinical Research Center for Oral Diseases, Department of Oral and Maxillofacial Surgery, West China Hospital of Stomatology, Sichuan University, Chengdu, China; ^2^State Key Laboratory of Oral Diseases and National Clinical Research Center for Oral Diseases, Department of Oral Pathology, West China Hospital of Stomatology, Sichuan University, Chengdu, China

**Keywords:** periodontitis, oral cancer, OSCC, periodontal pathogens, bacterial, periodontal disease

## Abstract

Emerging evidence shows a striking link between periodontal diseases and various human cancers including oral cancer. And periodontal pathogens, leading to periodontal diseases development, may serve a crucial role in oral cancer. This review elucidated the molecular mechanisms of periodontal pathogens in oral cancer. The pathogens directly engage in their own unique molecular dialogue with the host epithelium to acquire cancer phenotypes, and indirectly induce a proinflammatory environment and carcinogenic substance in favor of cancer development. And functional, rather than compositional, properties of oral microbial community correlated with cancer development are discussed. The effect of periodontal pathogens on periodontal diseases and oral cancer will further detail the pathogenesis of oral cancer and intensify the need of maintaining oral hygiene for the prevention of oral diseases including oral cancer.

## Introduction

Oral cancer (OC), predominantly oral squamous cell carcinoma (OSCC), accounted for almost 2.0% of all cancer cases and 1.9% of all cancer deaths globally from the report by the International Agency for Research on Cancer (IARC) in 2018 (Bray et al., 2018). Alcohol and tobacco assumptions are the foremost risk factors, however, cannot explain that the result of OC also commonly occurs in patients without exposure to alcohol or tobacco. Recently, infectious agents, researched as a significant role in the development and progression of OC, gradually come into view. The oral microbiome plays an essential role in the maintenance of normal oral physiology, and more attention has been currently given to the possible causality between the instabilities of microbiome dynamics and cancer. The role of bacterial infection in cancer initiation, promotion, and progression is firstly demonstrated by *Helicobacter pylori* (*H. pyloria*), a carcinogen of gastric cancer classified by IARC (Fox and Wang, 2007). *H. pyloria* is a dominant species of the human gastric microbiome, and the colonization of *H. pyloria* causes a persistent inflammatory response. *H. pyloria*-induced gastritis is the strongest singular risk factor for gastric cancer (Ranjbar et al., 2017). Present epidemiological data found that cancers are generally caused by the inflammatory response to bacterial infections. Some bacteria directly manipulate their host cell to affect the integrity and contribute to tumor formation in various phases of their infection cycle. Such as bacterial surface moieties, bacterial protein toxins, and bacterial effector proteins can induce host cell DNA damage, thereby interfering with essential signaling pathways involved in cancer cell development.

Periodontal disease (PD) is one of the most common inflammatory diseases in adults, predominantly caused by bacterial infection (Genco and Borgnakke, 2013). Most bacteria in the plaque are normal floras. However, a few bacteria in the plaque associated with diseased periodontal tissues have been identified as putative pathogens. Compared with patients without PD, mounting studies have reported a two- to five-fold increase in the risk of OC among those with PD (Javed and Warnakulasuriya, 2016; Shin et al., 2019). Studies also suggested that the causal relationship between the extent and severity of chronic periodontitis and the risk of OC is significant, even after the adjustments for traditional confound factors, i.e., smoking, alcohol, and human papillomavirus (HPV) (Moraes et al., 2016). It has been found that tooth loss as a result of bone loss in PD is an independent risk factor for head and neck cancer (Shi et al., 2018a). Also, high expression of the human telomerase reverse transcription, the expression of which is highly specific to cancer cells, was detected in patients with periodontitis (Katarkar et al., 2015). These all suggest that there is a striking link between PD and OC. Moreover, this link may be explained by the followings. (1) Broken mucosal barrier in PD consequently enhances penetration of carcinogens such as tobacco and alcohol. (2) Immunosuppression leads both to PD and OC. (3) Viruses such as HPV and *Candida albicans* are found both in PD and OC. (4) Chronic inflammation in PD contributes to cancer. (5) Dysbacteriosis in PD further leads to carcinogenic effects.

Furthermore, recent studies have confirmed that colonization of periodontal pathogens is a risk factor for OC independent of alcohol, smoking, and HPV (Ganly et al., 2019). It is interpreted to indicate that periodontal pathogens contribute to the link between PD and OC, and it would represent an obvious potential target for therapeutic intervention. Therefore, this review summarized the molecular mechanism of organisms and the production of carcinogenic substances and proinflammatory environment caused by pathogens, which shed light on the impact of periodontal pathogens on OC. The literature search was conducted through PubMed and Google Scholar. Research articles, published from 2000 to 2021, describing periodontitis, periodontal pathogens, PD, bacteria, cancer, and OC were selected.

## Association Between Periodontal Pathogens and Oral Cancer

It is well known the “red complex” has been proposed as a pathogenic consortium of PD, consisting of *Porphyromonas gingivalis* (*P. gingivalis*), *Tannerella forsythia* (*T. forsythia*), and *Treponema denticola* (*T. denticola*) (Holt and Ebersole, 2000). Also, mounting evidence has identified that more bacteria were detected from PD sites as causative periodontal pathogens, namely, *Aggregatibacter actinomycetemcomitans* (*A. actinomycetemcomitans*), *Fusobacterium nucleatum* (*F. nucleatum*), *Prevotella intermedia* (*P. intermedia*), *Streptococcus intermedius* (*S. intermedius*), *Prevotella tannerae* (*P. tannerae*), *Prevotella melaninogenica* (*P. melaninogenica*), *Prevotella intermedia* (*P. intermedia*), *Campylobacter recta* (*C. recta*), *Capnocytophaga gingivalis* (*C. gingivalis*), *Streptococcus mitis* (*S. mitis*), and so on (Nonnenmacher et al., 2001; Colombo et al., 2009). Furthermore, many other microbial pathogens have been detected in periodontal lesions apart from bacteria, namely, human cytomegalovirus, Epstein-Barr virus, HPV, and *Candida* (especially *C. albicans*), which all proved to be associated with the PD (Slots and Slots, 2000; Sardi et al., 2010).

Research data to date corroborated the significant positive association between PD (especially periodontitis) and total cancer risk, particularly for head and neck cancer, digestive tract cancer, pancreatic cancer, prostate cancer, breast cancer, lung cancer, hematological cancer, and lymphatic cancer (Corbella et al., 2018) (Table 1). That abnormal levels of periodontal pathogens detected in tissue samples from the patients with various forms of cancer suggested that periodontal pathogens serve a potentially crucial role in the development and progression of cancer.

**Table 1:** List of different types of cancer associated with periodontitis and possible associated periodontal pathogens.

**Cancer type**	**Research**	**Possible associated periodontal pathogens**
Oral cancer	Javed and Warnakulasuriya (2016); Shin et al. (2019)	*P. gingivalis, F. nucleatum, T. forsythia, P. intermedia, C. gingivalis, P. melaninogenica, S.mitis* (Nagy et al., 1998; Mager et al., 2005; Hu et al., 2016; Chang et al., 2019a)
Head and neck SCC	Tezal et al. (2009); Zeng et al. (2013)	*P. gingivalis, F. nucleatum, Actinomyces* (Mougeot et al., 2020; Metsäniitty et al., 2021)
Digestive tract cancer	Kim et al. (2019); Zhang et al. (2020)	*T. denticola, P. intermedia, Rothia, Prevotella* (Kato et al., 2016; Flemer et al., 2018; Yang et al., 2019)
Pancreatic cancer	Chang et al. (2016), Maisonneuve et al. (2017)	*P. gingivalis, A. actinomycetemcomitans* (Fan et al., 2018)
Prostate cancer	Lee et al. (2017a); Wei et al. (2020)	*P. gingivalis, T. denticola* (Estemalik et al., 2017) and so on
Lung cancer	Zeng et al. (2016); Wang et al. (2020)	*P. intermedia, C. rectus, F. nucleatum, Capnocytophaga* (Yan et al., 2015; Mai et al., 2016) and so on
Breast cancer	Sfreddo et al. (2017); Shi et al. (2018b)	*F. nucleatum* (Van der Merwe et al., 2021) and so on
Hematological cancer	Chung et al. (2016); Wu et al. (2020)	*Rothia, Actinomyces* (Mougeot et al., 2020) and so on
Non-hodgkin lymphoma	Bertrand et al. (2017); Wu et al. (2020)	Not mentioned

The role of periodontal pathogens in head and neck squamous cell carcinoma (HNSCC) particularly OSCC is a hotspot and keystone. A group of periodontitis-correlated taxa was detected in patients with OC. For instance, *Fusobacterium, Dialister, Peptostreptococcus, Filifactor, Peptococcus, Catonella*, and *Parvimonas* were significantly enriched in OSCC samples, and *Veillonella, Fusobacterium, Prevotella, Porphyromonas, Actinomyces, Clostridium, Haemophilus, Enterobacteriaceae*, and *Streptococcus* spp. were increased at tumor sites (Zhao et al., 2017; Zhang et al., 2019). Three bacterial species, *C. gingivalis, P. melaninogenica*, and *S. mitis* were elevated in the 80% saliva of individuals with OSCC and have been suggested as potential biomarkers for OC on account of a diagnostic sensitivity of 80% and a specificity of 82% (Mager et al., 2005). *P. gingivalis* and *F. nucleatum* were detected at higher levels in patients with OSCC tissues than in normal tissues (Chang et al., 2019a), and *F. nucleatum, P. intermedia*, and *P. tannerae* showed a significantly higher relative abundance in patients with OSCC compared with controls (Hsiao et al., 2018). Particularly, *P. gingivalis* infection was positively associated with late clinical staging, low differentiation, and lymph node metastasis in patients with OSCC (Chang et al., 2019a). Notably, for patients with OSCC, *C albicans* was detected at tumor sites, but never at control sites, which suggests that *C. albicans* has a property that is important in OC (Nagy et al., 1998). Growing studies provide supportive evidence that oral microbiota especially periodontal pathogens are involved in the development of OC. However, the direct causal effect of PD on OC, like *H. pyloria* infection is a pathogenic factor of gastric cancer, is still needed to explore.

## Mechanism of Periodontal Pathogens Leading to Cancer

**Figure 1:**
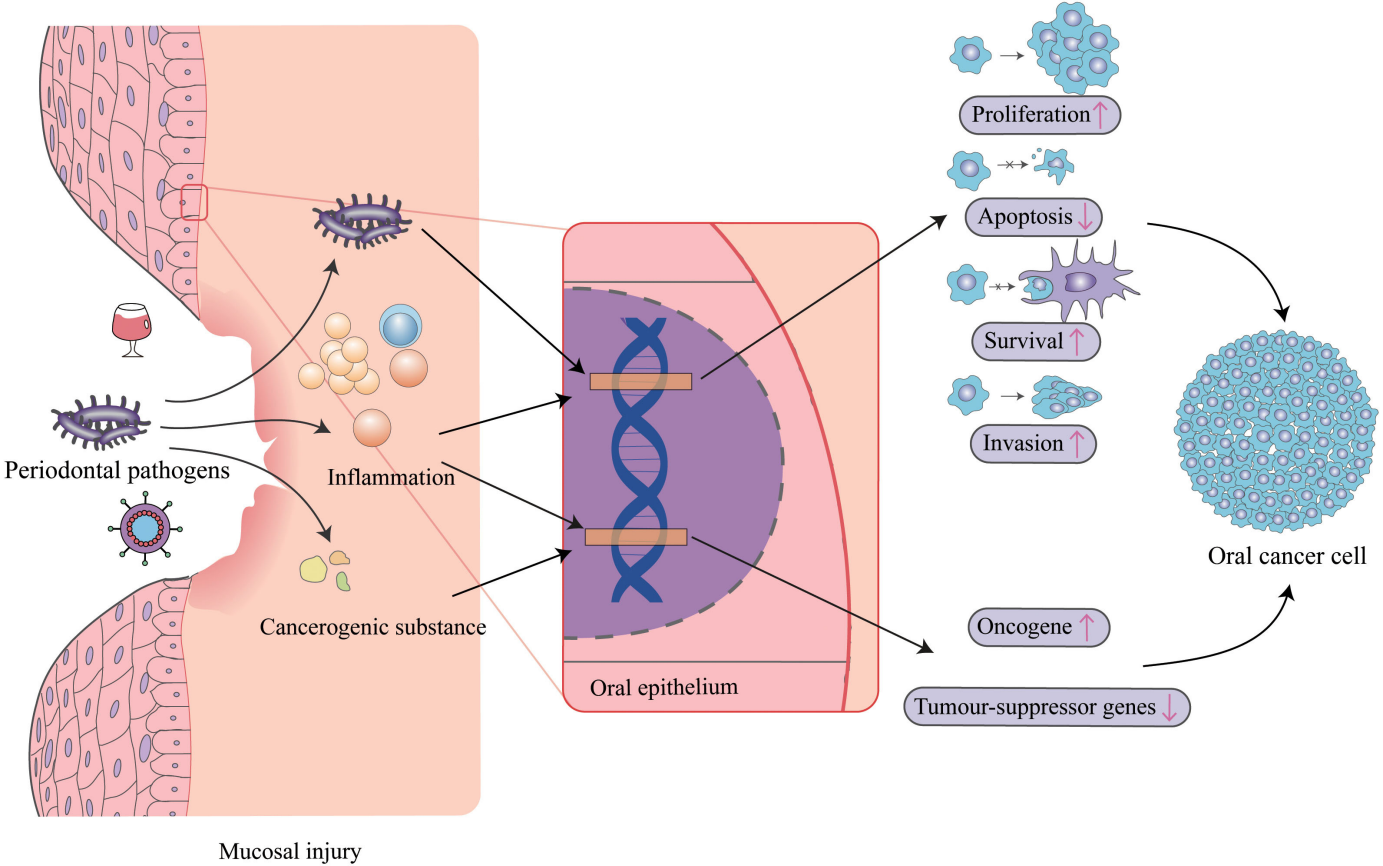
Mechanisms of periodontal pathogens impacting oral cancer. Periodontal pathogens can invade oral epithelial cells and directly impact target genes to cause changes in cell proliferation, apoptosis, survival, and invasiveness. Pathogens also triggers inflammation, which also affects these biological pathways, and similarly acts on oncogenes and tumor-suppressor genes, as do some cancerogenic substances produced by periodontal pathogens. In addition, inflammation sequentially induces mucosal injury leading to susceptibility to virus and other carcinogens like alcohol.

Periodontal pathogens have been proposed to induce carcinogenesis either through induction of chronic inflammation, interference with eukaryotic cell cycle and signaling pathways, or metabolism of potentially carcinogenic substances (Figure 1). Numerous studies demonstrated that some periodontal pathogens can affect specific intracellular pathways, promote cell survival, activate oncogenic pathways, reduce proapoptotic protein expression, and increase cell migration and invasion. Also, it is a fact not lost on the microbial community, which is thought to determine the potential for disease.

### Stimulation of Chronic Inflammation

Chronic or dysregulated inflammation has long been appreciated as a major contributor to tumor induction, progression, invasion, and metastasis, in part through modulation of the tumor microenvironment by cytokines, chemokines, prostaglandins, and reactive oxygen and nitrogen radicals accumulation in the microenvironment of tissues (Feller et al., 2013). These inflammatory factors, if persistent, have the capacity to induce cell proliferation and promote prolonged cell survival through activation of oncogenes and inactivation of tumor-suppressor genes. There is now a wealth of evidence indicating a link between chronic inflammation and malignant transformation of the affected oral epithelium (Tampa et al., 2018). And periodontal pathogenic bacteria (especially *P. gingivalis, P. intermedia, T. denticola*, and *F. nucleatum*) cause and maintain constant chronic inflammatory response, which induces the destruction of periodontal tissue and furthermore carcinoma development (Hajishengallis, 2015). The periodontal pathogens participate in osteoclastogenesis, collagen degradation, and alveolar bone resorption by secreting interleukins (ILs), which are members of cytokines that contribute to the immunological responses of many cells and tissues (Behzadi et al., 2016, 2022), tumor necrosis factor-alpha (TNF-α), matrix metalloproteinases (MMPs), and so on from inflammatory macrophages (Hienz et al., 2015). Furthermore, pathogens also impact oral carcinogenesis based on these increased levels of inflammatory factors after the initial inflammatory response (Table 2).

**Table 2:** List of some cytokines secreting by periodontal pathogens stimulation, the role of which in periodontitis and oral cancer and the signaling pathways involved in oral cancer.

**Type of cytokine**	**Pathogens**	**Signaling pathway in oral cancer**
IL-6	*P. gingivalis, T. forsythia, A. actinomycetemcomitans* (Yee et al., 2014; Cheng et al., 2016; Geng et al., 2019)	JAK-STAT3-SNAIL, MMP-1, MMP-9,TGF-β1, DNA hypomethylation, aberrant promoter hypermethylation (Sundelin et al., 2005; Gasche et al., 2011; Yadav et al., 2011)
IL-8	*P. gingivalis, F. nucleatum* (Yee et al., 2014; Casasanta et al., 2020)	MMP-1, MMP-2, MMP-10, IL-8/CXCL1 (Khurram et al., 2014; Ha et al., 2015, 2016)
IL-1β	*P. gingivalis, F. nucleatum, T. forsythia, A. actinomycetemcomitans* (Cheng et al., 2016)	IL-6, IL-8, CXCL1, NF-κB, EGFR (Lee et al., 2015a,b)
IL-17	*P. gingivalis, A. actinomycetemcomitans* (Cheng et al., 2016)	IL-23/IL-17, IL-8, IL-1β, TNF-αMCP-1, GRO-α, TGF-β, G-CSF, GM-CSF, IL-6/STAT3 (Xu and Cao, 2010; Gu et al., 2011)
IL-23	*P. gingivalis, A. actinomycetemcomitans* (Cheng et al., 2016)	IL-23/IL-17, IL-6, TNF-α, NF-κB (Caughron et al., 2018)
TNF-α	*P. gingivalis, F. nucleatum, A. actinomycetemcomitans* (Cheng et al., 2016; Abdulkareem et al., 2018)	MMP-1, MMP-9, MiR-21, miR-450a (Sundelin et al., 2005; Qiu et al., 2018; Hsing et al., 2019)
TGF-β1	*P. gingivalis, F. nucleatum* (Abdulkareem et al., 2018)	VEGF, HIF-1α, MMP-9, IL-6 (Chen et al., 2012)
EGF	*P. gingivalis, F. nucleatum* (Abdulkareem et al., 2018)	Warburg effect, EGFR/PI3K/HIF-1α, CD206, miR-31, IMP-3, PI3K/AKT/WNT7A/β-catenin/MMP9 (Lu et al., 2014; Zhang and Jung, 2016; Xu et al., 2017; Haque et al., 2019; Xie et al., 2020)
CXCL1/ GRO-α	*F. nucleatum* (Yu et al., 2017)	IL-8/CXCL1, EGFR (Zhang et al., 2010; Zeng et al., 2013)

In OSCC cells, *P. gingivalis* stimulates the release of a variety of chemokines and cytokines contributing to cancer, namely, IL-1β, IL-6, IL-8, TGF-β1, EGF, and TNF-α (Yee et al., 2014; Abdulkareem et al., 2018). In addition, *P. gingivalis* and *A. actinomycetemcomitans* can activate monocytes resulting in increased IL-17 production by human CD4+ T cells *in vitro*, a process that appears to have enhanced in patients with PD (Cheng et al., 2016), and IL-23/IL-17 pathway is proved promotive in tumorigenesis (Grivennikov et al., 2012). *F. nucleatum* increases the secretion of IL-1β *via* activation of the NOD-, LRR-, and pyrin domain-containing protein 3 (NLRP3) inflammasome and caspase 1 to induce nuclear localization of NF-κB in gingival epithelial cells (GECs) (Bui et al., 2016). Moreover, *F. nucleatum* can induce an epithelial-to-mesenchymal transition (EMT) process in OSCC cells through upregulation of TGF-β, TNFα, and EGF signaling (Abdulkareem et al., 2018). *T. forsythia* can induce pro-inflammatory cytokines such as IL-1β and IL-6 by CD4 + T helper cells and TNF-α in esophageal squamous cell carcinoma (ESCC) (Malinowski et al., 2019). In conclusion, inflammatory mediators, at least partly, regulated by periodontal pathogens during PD development may mediate oral malignant transformation, but the underlying mechanism still remains unclear.

### Metabolic By-Products Contributing to Carcinogenesis

The microflora existing in the tumor microenvironment may aid in tumorigenesis for some of its metabolic derivatives being able to induce damage to the DNA, mutagenesis, and secondary hyperproliferation of the oral cells. Acetaldehyde, a metabolite of ethanol, has been proved to be carcinogenic in both animal models and *in vitro* studies. It has been shown that some *Neisseria*-strains (Muto et al., 2000; Tagaino et al., 2019) and *Streptococcus*-strains (Kurkivuori et al., 2007; Tagaino et al., 2019) (especially viridans group *streptococci*, namely, *Streptococcus salivarius, S. intermedius*, and *S. mitis*) metabolize ethanol to carcinogenic acetaldehyde in saliva by the alcohol dehydrogenase. In addition, *Rothia mucilaginosa* and *Prevotella histicola* also exhibit the ability to produce acetaldehyde (Moritani et al., 2015). And *C. albicans*-strains also have earlier been shown to be massive acetaldehyde producers (Tillonen et al., 1999).

Hydrogen sulfide (H_2_S), a gasotransmitter exerting important physiological and pathological functions in the entire body, can be produced by some oral bacteria including periodontal pathogens *T. denticola* and *P. gingivalis* (Persson et al., 1990). Considering that H_2_S represents an index of oral hygiene, it is thought to be associated with oral diseases including PD and OC (Zhang et al., 2010). Zhang et al. for the first time demonstrated that H_2_S promotes OC cell proliferation through the COX2/AKT/ERK1/2 axis (Zhang et al., 2016). However, the underlying mechanisms regulating the multiple functions of H_2_S in many tissues and organs remain unknown.

Nitrosamine, considered a potential carcinogen, can be produced by commensal bacteria and *Candida* spp. (Calmels et al., 1988). Such a carcinogen can induce point mutations leading to activating specific oncogenes and initiating the development of OC (Oliveira et al., 2007). Some *Candida* spp. were found to be able to produce the potent carcinogen N-nitrosobenzylmethylamine (NBMA), and strains with the highest potential to produce NBMA were isolated from advanced, potentially malignant, oral mucosal lesions rather than early lesions or normal oral mucosa (Krogh et al., 1987). The tubular hyphal structure of *C. albicans* allows ingress of precursors from saliva and release of the nitrosamine product to keratinocytes, potentially initiating OSCC (Dwivedi et al., 2009).

Free fatty acid, production of fatty acid metabolism, may contribute to oral carcinogenesis. Wu et al., first, demonstrated that *P. gingivalis* was involved in fatty acid metabolism of oral carcinogenesis (Wu et al., 2018). They established a combined experimental system of 4 nitroquinoline 1-oxide (4NQO)-induced oral carcinoma model and *P. gingivalis*-treated chronic periodontitis model, and it has been found *P. gingivalis*-treated mice developed more and larger tumors in the tongue as compared with the carcinogen-alone group. It showed that the level of free fatty acid was significantly increased in the tongue and liver tissues of 4NQO-treated mice infected with *P. gingivalis*. This supports the previous speculation that cancer cells can utilize circulating free fatty acid from their microenvironment, a favorable microenvironment for tumorigenesis by fueling cancer cell survival and proliferation. These results indicate a close association between *P. gingivalis*, lipid metabolism, and oral carcinogenesis, however, the underlying molecular mechanism between them still remains unclear.

### Promotion of Cell Proliferation

Some genes that control normal cellular growth and proliferation are altered by exposure to exogenous or endogenous mutagens, subsequently causing clonal growth of the resulting precancerous or cancerous cells. Periodontal pathogens can perturb diverse pathways that constrain the proliferative response in normal cells in most cancers.

FimA, the fimbrial protein of *P. gingivalis*, can accelerate the progression of primary GECs through the S-phase of the cell cycle by manipulation of cyclin/cyclin-dependent kinases (CDKs) activity, reducing the level and the activity of the p53 tumor suppressor and increasing levels of phosphoinositide 3-kinase (PI3K) and phosphoinositide-dependent protein-serine kinase 1 (PDK1) (Kuboniwa et al., 2008). In OSCC cells, *P. gingivalis* regulates cyclin D1 expression through the miR-21/PDCD4/AP-1 negative feedback signaling pathway to increase cell proliferation (Chang et al., 2019b). The exposure of oral epithelial cells to *P. gingivalis and F. nucleatum* triggers Toll-like receptors, pivotal biomolecules in the immune system (Behzadi et al., 2021). It may result in IL-6 production that activates STAT3 which in turn induces cyclin D1 driving OSCC growth. *P. gingivalis* infection diminishes both the level and the activity of p53, consistent with an increased proliferation rate of infected GECs (Kuboniwa et al., 2008). *P. gingivalis* infection increases levels of PI3K and PDK1 (a key molecule that couples PI3K to cell proliferation and survival signals) (Kuboniwa et al., 2008). Consistent with this, phosphatase and tensin homolog (PTEN), a lipid phosphatase negatively regulating the PI3K pathway, was downregulated and inactivated by phosphorylation after *P. gingivalis* infection (Yilmaz et al., 2004; Kuboniwa et al., 2008).

Besides, pathogens can impact β-catenin signaling, a major pathway contributing to the control of cell proliferation and tumorigenesis. Gingipain, a cell surface proteinase of *P. gingivalis*, plays a key role in the β-catenin process. *P. gingivalis* induces the activation of β-catenin and the disassociation of the β-catenin destruction complex by the gingipain-dependent proteolytic process (Zhou et al., 2015). Processed β-catenin can be translocated to the nucleus, where it binds the TCF/LEF promoter element, and finally stimulates the expression of Myc and cyclin D1 (Zhou et al., 2015). Adhesin FadA, a virulence factor identified from *F. nucleatum*, is thought to play a major role in colorectal cancer (CRC) by binding to E-cadherin on CRC cells to activate β-catenin signaling (Rubinstein et al., 2013). The FadA-E-cadherin axis also upregulates annexin A1, a modulator of Wnt/β-catenin-based proliferative signaling in CRC cells (Rubinstein et al., 2019). And in OSCC, the research found that *F. nucleatum* infection promotes the proliferation ability of tongue squamous cell carcinoma cells by causing DNA damage *via* the Ku70/p53 pathway (Geng et al., 2020). Ku70 and p53 are both major proteins involved in regulating nonhomologous end-joining (NHEJ) repair, which is the most common DNA double-strand break (DSB) repair pathway in mammalian cells to prevent malignant transformation (Mari et al., 2006).

In addition, *P. gingivalis* can increase the gene expression of α-defensins, which have been found to exert multiplying effects on OC cell proliferation *via* direct epidermal growth factor receptor (EGFR) in dermal keratinocytes activation (Hoppe et al., 2016). However, *A. actinomycetemcomitans* is able to enhance cell death, which performed an opposite effect on cancer cell proliferation behavior (Hoppe et al., 2016).

Up to now, the impact of *P. gingivalis* infection on host cell proliferation remains controversial. Although most studies found that *P. gingivalis* infection promotes cancer cell proliferation and further contributes to carcinogenicity, some investigators observed that *P. gingivalis* inhibits cancer cell proliferation *via* inducing their apoptosis (Cho et al., 2014). The researchers demonstrated that *P. gingivalis* suppresses cell proliferation through G1 arrest in OC cells by inducing autophagy activated by the formation of reactive oxygen species (Cho et al., 2014). This may be caused by various complicated factors in the experiment, so more normalized studies and more solid evidence are necessary for elucidating the roles of pathogenic bacteria in cancer cell proliferation.

### Inhibition of Cell Apoptosis

Apoptosis is a distinct mode of cell death that is responsible for the deletion of cells in normal tissues. It can destroy the disrupted cell and prevent it from developing into a malignant tumor (Lowe and Lin, 2000; Behzadi and Behzadi, 2006). Thus, any agent capable of impeding apoptosis would promote the atypical build-up of cancerous cells. There have been several evidence of oral pathogens suppressing apoptosis and potentially promoting carcinogenesis. *P. gingivalis* activates the Jak1/Akt/Stat3 signaling to control intrinsic mitochondrial apoptosis pathways (Yilmaz et al., 2004; Yao et al., 2010). At the mitochondrial membrane, the activity of proapoptotic effectors such as Bad is inhibited, and the ratio of antiapoptotic factor Bcl2 to proapoptotic factors Bax is enhanced as well, which consequently curtails the discharge of the apoptosis effector cytochrome C (Yao et al., 2010).

A *P. gingivalis* homolog of nucleoside diphosphate kinase (NDK), a bacterial effector, is secreted extracellularly and serves a variety of cellular housekeeping functions such as DNA cleavage/repair, transcriptional regulation, cell proliferation, and apoptosis (Yu et al., 2017). *P. gingivalis* can inhibit GEC apoptosis induced by ATP ligation of purinergic receptor P2X_7_, and this effect is mediated by NDK (Yilmaz et al., 2008). Another antiapoptotic function of *P. gingivalis*-NDK is phosphorylating heat-shock protein 27 (HSP27) in GECs, which curtails cytochrome C release and caspase 9 activation (Lee et al., 2018).

Forkhead box-O (FOXO)1, one of the forkhead transcription factors, controls oxidative stress responses, inflammatory cytokine production, and cell survival. *P. gingivalis* induces the dephosphorylation and activation of FOXO1, while FOXO1 knockdown can impede *P. gingivalis*-induced antiapoptosis gene transcription (Wang et al., 2015). *P. gingivalis*-FimA, targeting chemokine receptor type 4 (CXCR4), plays a primary role in promoting OSCC tumor growth through the phospho-Akt1 (pAKT1)-pFOXO1-dependent pathway (Arjunan et al., 2018). Intriguingly, this apoptosis-resistant pathway also involves immunosuppression through the induction of myeloid-derived dendritic suppressor cells (MDDSCs), predominantly dependent on the dendritic cell-specific intercellular adhesion molecule-3-grabbing nonintegrin (DC-SIGN) targeting Mfa1 fimbriae of *P. gingivalis* (Arjunan et al., 2018). It suggests that Mfa1, high expression in chronic periodontitis samples (Arjunan et al., 2018), may be involved in immunosuppression in the pathogenesis of periodontitis through the pAKT1-pFOXO1 pathway. Phosphorylated FOXO1 also regulates FOXP3 expression through its feedback regulatory loop mechanism as per the consistent and continuous stimulation of *P. gingivalis* strains in MDDSCs, to promote apoptosis resistance and immunosuppression (Arjunan et al., 2018).

Upregulated expression of miR-203 induced by *P. gingivalis* inhibits the suppressor of cytokine signaling 3 signaling and increases Stat3 activation, and then inhibits apoptosis and accelerates cell cycle progression in GECs (Moffatt and Lamont, 2011).

As previously mentioned, oral pathogens like *P. gingivalis* exhibit both proapoptotic and antiapoptotic phenotypes, which may be depending on contextual and temporal cues (Byrne and Ojcius, 2004). For instance, apoptosis can be induced in some cell types by *P. gingivalis* components such as proteases, whereas other cellular constituents such as fimbriae and lipopolysaccharide can either suppress or induce apoptosis depending on the host cell type. Also, the invasion of metabolically active *P. gingivalis* can favor host cell survival, in contrast to the apoptotic effects induced by heat-killed noninvasive *P. gingivalis*.

### Promotion of Cell Survival

Periodontal pathogens may enhance the survival of tumor cells through some approaches of intracellular or extracellular mechanisms apart from inhibiting apoptosis.

**Figure 2:**
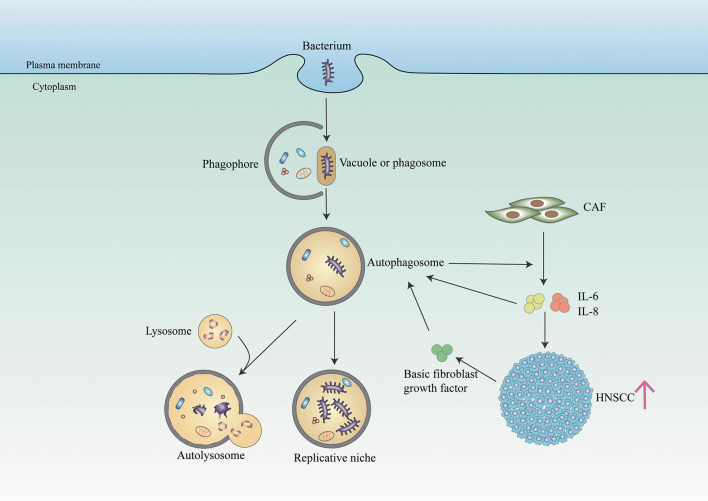
Bacteria and autophagy interplay in cancer cells. The bacterium (such as *P. gingivalis*) resides in a bacterium-containing vacuole (or phagosome) after invasion of host cells. Phagophore is assembled and starts to elongate to enclose cytoplasmic components on the stimulation of autophagy to form autophagosome. And autophagosome serves as a replicative niche in which they are not eliminated. In addition, some bacterium toxicity is degraded in the autolysosome generated by fusing autophagosome and lysosome. In NHSCC, autophagy regulates the secretion of IL6 and IL8 from CAFs, facilitating HNSCC migration. And paracrine secretion of IL6, IL8, and basic fibroblast growth factors promotes CAF autophagy, which is further maintained through IL6 and IL8 autocrine feedback.

Autophagy, an intracellular catabolic process, serves to capture and degrade intracellular components for homeostasis. Patients with periodontitis presented a higher level of autophagy activity compared with patients in a healthy periodontal state (Wei et al., 2018). It has been suggested that autophagy protects periodontal cells from apoptosis, promotes angiogenesis, and facilitates oral bacteria like *P. gingivalis* to escape from the host's responses (Wei et al., 2018). Similarly, in cancer including OC, the autophagy process is also upregulated and promotes cancer cell survival (Mathew et al., 2007) (Figure 2). Recent studies demonstrated that OC cells promote autophagy as an adaptive mechanism against the invasion of bacteria bys limiting the toxicity and helping cancer cells to survive (Huang and Brumell, 2014). New et al. found that autophagy-dependent secretion of tumor-promoting factors, notably IL6 and IL8, secreted by HNSCC-associated cancer-associated fibroblasts (CAFs) contributes to the malignant development of HNSCC (New et al., 2017). And Chen et al. showed that autophagy activation may contribute to the elevated IL-6 production in *P. gingivalis*-infected ESCC cells, which promotes esophageal cancer development and progression (Chen et al., 2021). Besides, *F. nucleatum* promotes metastasis in CRC by activating autophagy signaling *via* the upregulation of CARD3 expression (Chen et al., 2020). However, some studies found the role of autophagy in promoting cancer is controversial, which requires further studies to elaborate on the relationship between autophagy and periodontal pathogens in OC (Levy et al., 2017).

Immune evasion is another approach for pathogens to promote cancer cell survival. In squamous carcinoma cells, *P. gingivalis* can induce the expression of programmed death-ligand 1 (PD-L1, B7-H1) and B7-DC receptors functioning anergy and apoptosis of activated T cells, which enable tumor cells to overcome host response (Groeger et al., 2011). *F. nucleatum* can also protect tumors from immune cell attack, by activating the Fap2 (an adhesion of *F. nucleatum)*-dependent inhibitory immunoreceptor T cell immunoglobulin, ITIM domain (TIGIT), and carcinoembryonic antigen cell adhesion molecule 1 (CEACAM1) to suppress the activities of T and natural killer cells (Gur et al., 2015, 2019).

Periodontal pathogens also assist resistance of cancer cells to chemotherapeutic reagents of oral squamous cell carcinoma. Tumor xenografts composed of *P. gingivalis*-infected OSCC cells exhibited higher resistance to Taxol through Notch intracellular domain 1 activation (Woo et al., 2017), and a higher serum level of IL-6 was detected compared with uninfected mice (Song et al., 2019). It suggested that *P. gingivalis* might play a role in the development of chemoresistance toward OSCC. Intriguingly, researchers discovered that targeting Notch signaling pathways and prophylactic use of anti-inflammatory drugs (such as ibuprofen) may be used to overcome drug resistance to cancer therapy (Wang et al., 2010; Woo et al., 2017; Song et al., 2019).

### Promotion of Cell Invasion

Epithelial-to-mesenchymal transition (EMT) is one of the vital processes of cancer malignancy through the loss of its morphology from epithelial cell types to the morphology of mesenchymal cell types. The process is executed by so-called EMT-activating transcription factors, mainly of the SNAIL, TWIST, and ZEB families. *P. gingivalis* initiates EMT through FimA-driven ZEB1 expression in GECs, which provides a mechanistic basis for the *P. gingivalis* contribution to OSCC, and *P. gingivalis* retained the capacity to upregulate ZEB1 when co-infected with either species like *S. gordonii* or *F. nucleatum* (Sztukowska et al., 2016). Recently, Qi et al. found that *P. gingivalis* promotes EMT and stemness features of ESCC *via* TGFβ-dependent *Drosophila* mothers against decapentaplegic homologs (Smads)/yes-associated protein (YAP)/transcriptional coactivator with PDZ-binding motif (TAZ) signaling (Qi et al., 2020). Prolonged and repetitive exposure to *P. gingivalis* infection induced acquisition of stemness that was indicated by increased expressions of both CD44 and CD133 and tumor sphere-forming ability (Ha et al., 2015). And *P. gingivalis* infection promotes cell migration, which was slightly enhanced by co-infection with *F. nucleatum* (Lee et al., 2017b). *F. nucleatum* can induce an EMT program in OSCC cells by activation of Snail *via* TGF-β, tumor necrosis factor-α (TNF-α), and EGFR signaling, with upregulation of MMP-2, MMP-3, and MMP-9 (Abdulkareem et al., 2018).

Matrix metalloproteinases (MMPs), a family of zinc-dependent proteolytic enzymes, promote carcinoma cell migration and invasion and also play a major role in periodontal tissue destruction. *P. gingivalis* has been reported to upregulate the production of several MMPs, namely, MMP-1, MMP-2, MMP-7, MMP-9, and MMP-10, from primary and transformed oral epithelial cells (Inaba et al., 2014; Ha et al., 2015, 2016; Sztukowska et al., 2016; Lee et al., 2017b). These MMP productions are proved influenced by *P. gingivalis*-induced IL-8 (Ha et al., 2015, 2016). In the OSCC cellular invasion mechanism, *P. gingivalis* induces MMP-9 proenzyme expression through ERK1/2-Ets1, p38/HSP27, and PAR2/NF-kB pathways, after which the proenzyme is activated by gingipains (Inaba et al., 2014). *F. nucleatum* can increase the section of MMP-9 and MMP-13 through the activation of mitogen-activated protein kinase p38 and promote cellular migration possibly *via* stimulation of Etk/BMX, S6 kinase p70, and RhoA kinase (Uitto et al., 2005). Dentilisin, a chymotrypsin-like proteinase of *T. denticola* was found to convert pro-MMP-8 and−9 into their active forms and was able to degrade the proteinase inhibitors TIMP-1, TIMP-2, α-1-antichymotrypsin, and complement C1q, which contributes to an overall more proteolytic environment favoring invasion of epithelial cells (Nieminen et al., 2018).

### Oral Microbial Community Perturbations

**Figure 3:**
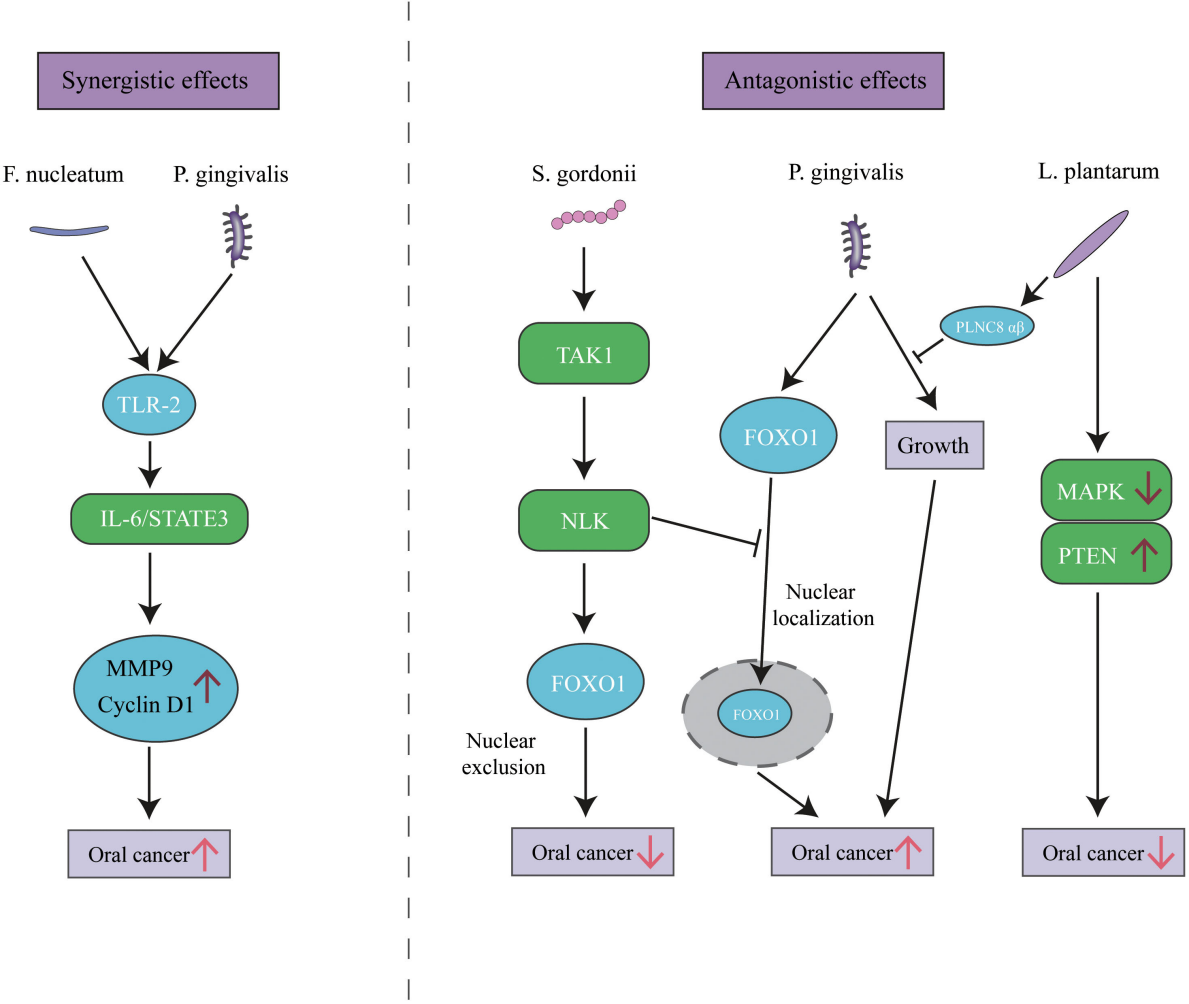
The synergistic and antagonistic effects among oral bacteria in oral cancer. *P. gingivalis* and *F. nucleatum* triggers TLR signaling, resulting in IL-6 production that activates STAT3 which in turn induces oral cancer growth and invasiveness. *P. gingivalis* can localize FOXO1 in nuclear to promote oral cancer, while the presence of *S. gordonii* can activate the TAK1-NLK1 pathway, which supersedes the effect of *P. gingivalis* and translocates FOXO1 to the cytoplasm, where it is inactive. *L. plantarum* can inhibit oral cancer through upregulation of PTEN and downregulation of MAPK pathways, and its bacteriocin PLNC8 αβ can suppress *P. gingivalis* growth and subsequent pathogenicity.

As with PD, it is likely that the communities rather than individual species serve the pathogenic role in OC. Interactions among bacterial components of the community can be synergistic and antagonistic. The promotion of OC progression by *P. gingivalis* can be slightly enhanced by co-infection with *F. nucleatum* (Lee et al., 2017b). However, *P. gingivalis*-induced cell migration is antagonized by S*treptococcus gordonii* (*S. gordonii*) through the TAK1-NLK negative regulatory pathway (Ohshima et al., 2019). Similarly, numerous antagonistic cases have been reported among oral bacteria. For instance, *Lactobacillus plantarum* (*L. plantarum)*, a part of the normal flora of humans, can inhibit *P. gingivalis* growth (Pudgar et al., 2021). *L. plantarum* can also inhibit OC development by inducing apoptosis in OC cells by upregulation of PTEN and downregulation of mitogen-activated protein kinases (Asoudeh-Fard et al., 2017) (Figure 3). The complex and diverse interactions within the polymicrobial communities perturb host homeostasis, which leads to diseases like PD or OC. Furthermore, Yost et al. performed a pilot metatranscriptomic analysis of the oral microbiome associated with human OSCC sites, and they found clear changes in microbial metabolic activities in OSCC, regardless of the community composition. These metabolic activities include iron acquisition, response to oxidative stress, and peptidase activity (Yost et al., 2018). It illustrates that metabolic activities are better correlated with disease than community microbial composition. Similarly, Perera et al. further revealed that more consistent informative results would be obtained with functional rather than compositional analysis (Perera et al., 2018). Available extent of the involvement of the oral microbiome in cancer represents only the tip of the iceberg, and the function of whether an individual bacterium or microbial community requires further disclosure.

## Conclusion

Since many epidemiological studies reveal a link between PD and OC, the involvement of periodontal pathogens is well recognized as a keystone. Available data suggest that periodontal pathogens may contribute to cancer progression (including cell survival, proliferation, apoptosis, and invasion) by both the direct bacterial effect and the indirect inflammatory response and metabolic carcinogen. It found that early undetected cancer or precancerous lesions facilitate the colonization and growth of oral bacteria to promote tumor progression further, which suggests that the dentist should consider the patient with PD as a high risk for malignancy. However, there have been some other data showing some pathogens also suppress tumor growth, so how to balance and leveraging the role of different bacteria in cancer will be conducive to better prevention and management of cancer. And the theory that it is a microbial community, not individual species, that is reasonable for cancer development and procession is gradually accepted, but the mechanisms behind this organized and precise community still need further study.

## Author Contributions

T-JL: conceptualization, investigation, and writing—original draft and visualization. Y-hH: investigation and writing-original draft. X-hL: conceptualization, project administration, funding acquisition, resources, and writing—review and editing. Y-lT: conceptualization, funding acquisition, supervision, and writing—review and editing. All authors contributed to the article and approved the submitted version.

## Funding

This work was supported by the National Natural Science Foundation of China grants (Nos. 82073000 and 81972542), the Clinical Project of West China College of Stomatology, Sichuan University (LCYJ2019-8), and the Exploration and research projects of West China College of Stomatology, Sichuan University (LCYJ2020-YJ-1).

## Conflict of Interest

The authors declare that the research was conducted in the absence of any commercial or financial relationships that could be construed as a potential conflict of interest.

## Publisher's Note

All claims expressed in this article are solely those of the authors and do not necessarily represent those of their affiliated organizations, or those of the publisher, the editors and the reviewers. Any product that may be evaluated in this article, or claim that may be made by its manufacturer, is not guaranteed or endorsed by the publisher.
